# Comparison of Joint and Muscle Biomechanics in Maximal Flywheel Squat and Leg Press

**DOI:** 10.3389/fspor.2021.686335

**Published:** 2021-08-05

**Authors:** Maria Sjöberg, Hans E. Berg, Lena Norrbrand, Michael S. Andersen, Elena M. Gutierrez-Farewik, Patrik Sundblad, Ola Eiken

**Affiliations:** ^1^Division of Environmental Physiology, Swedish Aerospace Physiology Centre, School of Engineering Sciences in Chemistry, Biotechnology, and Health (CBH), KTH Royal Institute of Technology, Stockholm, Sweden; ^2^Department of Orthopaedic Surgery, Karolinska University Hospital, Division for Orthopaedics and Biotechnology, CLINTEC, Karolinska Institutet, Stockholm, Sweden; ^3^Department of Materials and Production, Aalborg University, Aalborg, Denmark; ^4^KTH MoveAbility Lab, Deptartment of Engineering Mechanics, KTH BioMEx Centre, KTH Royal Institute of Technology, Stockholm, Sweden; ^5^Division of Clinical Physiology, Karolinska University Hospital, Department of Laboratory Medicine, Karolinska Institute, Stockholm, Sweden

**Keywords:** closed kinetic chain exercise, strength training, gravity-independent, eccentric overload, musculoskeletal model

## Abstract

The aim was to compare the musculoskeletal load distribution and muscle activity in two types of maximal flywheel leg-extension resistance exercises: horizontal leg press, during which the entire load is external, and squat, during which part of the load comprises the body weight. Nine healthy adult habitually strength-training individuals were investigated. Motion analysis and inverse dynamics-based musculoskeletal modelling were used to compute joint loads, muscle forces, and muscle activities. Total exercise load (resultant ground reaction force; rGRF) and the knee-extension net joint moment (NJM) were slightly and considerably greater, respectively, in squat than in leg press (*p* ≤ 0.04), whereas the hip-extension NJM was moderately greater in leg press than in squat (*p* = 0.03). Leg press was performed at 11° deeper knee-flexion angle than squat (*p* = 0.01). Quadriceps muscle activity was similar in squat and leg press. Both exercise modalities showed slightly to moderately greater force in the vastii muscles during the eccentric than concentric phase of a repetition (*p* ≤ 0.05), indicating eccentric overload. That the quadriceps muscle activity was similar in squat and leg press, while rGRF and NJM about the knee were greater in squat than leg press, may, together with the finding of a propensity to perform leg press at deeper knee angle than squat, suggest that leg press is the preferable leg-extension resistance exercise, both from a training efficacy and injury risk perspective.

## Introduction

Squat and leg press are two multi-joint lower-limb resistance exercises frequently used in sports conditioning (Escamilla et al., 2001; Schoenfeld, 2010) and rehabilitation (Escamilla et al., 1998), as well as to prevent musculoskeletal deconditioning during prolonged space flights (Cotter et al., 2015; Petersen et al., 2016). Generally, squat and leg press are performed using free weights or weight stacks, providing a uniform constant load, but they can also be performed using a flywheel exercise device, providing a varying exercise load (Alkner and Bring, 2019).

The gravity-independent flywheel exercise device was first developed for use in space to counteract muscle loss in astronauts during long-duration flights (Berg and Berg, 1993). The flywheel is mounted on a shaft, which is connected to a strap, and the external exercise load is generated by setting a flywheel into rotation by pulling the strap (Berg and Tesch, 1994). The flywheel inertia allows generation of maximal voluntary force throughout the concentric action, since the force applied to the strap is transformed to kinetic energy of the flywheel. When fully unwound, the strap rewinds in the opposite direction, and by decelerating the flywheel rotation over a short time in the last part of the eccentric phase, it is possible to produce eccentric overload (Norrbrand et al., 2010; Fernandez-Gonzalo et al., 2014).

Eccentric muscle actions have been shown to be key elements for training-induced muscle hypertrophy (Hather et al., 1991) and strength increase (Dudley et al., 1991; Norrbrand et al., 2008). Furthermore, eccentric muscle work is essential in many sports-related movements including sprints, jumps and side-cut manoeuvres (Vogt and Hoppeler, 2014), and alpine skiing turns (Berg et al., 1995). It has been reported that the muscle hypertrophy and strength increases induced by flywheel resistance training are comparable, or even greater, than those induced by conventional weight training (Alkner and Tesch, 2004; Norrbrand et al., 2008; Maroto-Izquierdo et al., 2017; Petré et al., 2018). Presumably, a more efficient resistance training can be accomplished if the eccentric load can be maximised, as in flywheel exercise, rather than being restricted at a submaximal level dictated by the concentric muscle strength, as in conventional weight lifting (cf. Norrbrand et al., 2008; Petré et al., 2018; Carroll et al., 2019; Beato and Dello Iacono, 2020). Other reasons for a growing interest in flywheel-based resistance training in athletes, include reports of increased linear sprint (de Hoyo et al., 2015) and change-of direction speed (Tous-Fajardo et al., 2016), as well as reduced muscle injuries (Askling et al., 2003), and reduced severity of muscle injuries (de Hoyo et al., 2015), in soccer players performing flywheel resistance training.

To optimise training regimens while minimising the risk of inflicting load-dependent injuries, it is important to quantify the muscle use and load distribution encountered during near-maximal efforts of the specific exercise. Inverse dynamics combined with musculoskeletal modelling have proven powerful tools to evaluate muscle effort and joint loading, especially for complex multi-joint exercises with muscle activations that are difficult to predict (Chiu, 2018). This method has been used to study conventional barbell squat (Wretenberg et al., 1993; Escamilla, 2001; Schoenfeld, 2010; Bryanton et al., 2012), and to a lesser degree, leg press (Wilk et al., 1996; Escamilla et al., 2001; Kolditz et al., 2015). Comparisons of joint loads between these two exercises have, however, almost exclusively focused on the knee joint (Wilk et al., 1996; Escamilla et al., 1998, 2001). To date, only two studies have investigated joint biomechanics during flywheel resistance exercise: Chiu and Salem (2006) compared joint kinetics between flywheel and barbell front squats, and recently our group published a study comparing joint loads during submaximal flywheel squat and leg press (Sjöberg et al., 2020). However, corresponding joint loads and muscle forces during maximal flywheel squat and leg press have yet not been compared.

Consequently, the objective of the present study was to compare lower extremity joint kinematics, kinetics and muscular effort during maximal flywheel leg press and flywheel squat from training efficacy and injury risk perspectives. Based on findings obtained during submaximal flywheel exercise (Sjöberg et al., 2020), we hypothesised that leg press would produce higher moments than squat about the hip and ankle but not about the knee joint, and therefore, that leg press would activate the hip-extensor but not the knee-extensor muscles to a greater extent than would squat.

## Materials and Methods

### Experiment Design

The contributions of hip extensors, knee extensors and ankle-plantar flexors during maximal flywheel leg press and flywheel squat were studied in recreationally trained adults. Following one familiarisation session, the subjects participated in an experimental session in which kinematic and kinetic data were recorded using motion analysis. Lower-extremity joint angles, net-joint moments (NJM), muscle forces and muscle activities were subsequently computed from the experimental data using a musculoskeletal model and were then compared between the exercises using a within-subjects design.

### Subjects

Nine healthy individuals (6 women and 3 men, Table 1) volunteered to participate in the study and provided written consent after being informed of study aims and potential risks. Upon enrolment, they were unfamiliar with flywheel exercise but had regularly been performing conventional strength training involving the lower extremities, i.e., at least once a week during more than 6 months prior to the study, and were thus accustomed to free-weight squat and leg press exercises. The subjects were healthy and reported no previous or present injury that restricted them from performing the exercises properly.

**Table 1:** Description of individuals.

**Subject**	**Body height (m)**	**Body weight (kg)**	**Age (yrs)**	**Gender**	**Isometric quadriceps strength (BW)**
1	1.67	61.9	30	Female	1.76
2	1.70	54.9	25	Female	2.03
3	1.65	61.2	26	Female	2.62
4	1.63	59.4	27	Female	1.76
5	1.70	68.6	24	Female	2.44
6	1.74	78.6	27	Male	2.03
7	1.72	63.4	25	Female	2.04
8	1.80	77.8	21	Male	2.12
9	1.73	73.9	24	Male	2.98

*Isometric quadriceps strength is normalised to body weight in newtons (BW)*.

### Procedures

During the familiarisation session, submaximal flywheel repetitions were preceded by a warm up. Each subject performed several sets of submaximal and maximal repetitions in both configurations (leg press and squat; see further below), until using a proper technique. During the familiarisation session, each subject also performed three maximal isometric quadriceps contractions, while seated in the “leg-press position” on the flywheel device and with hip and knee angles being restricted to 90°. The 1-sec peak of the highest recorded force value was defined as the isometric maximum (Table 1).

**Figure 1:**
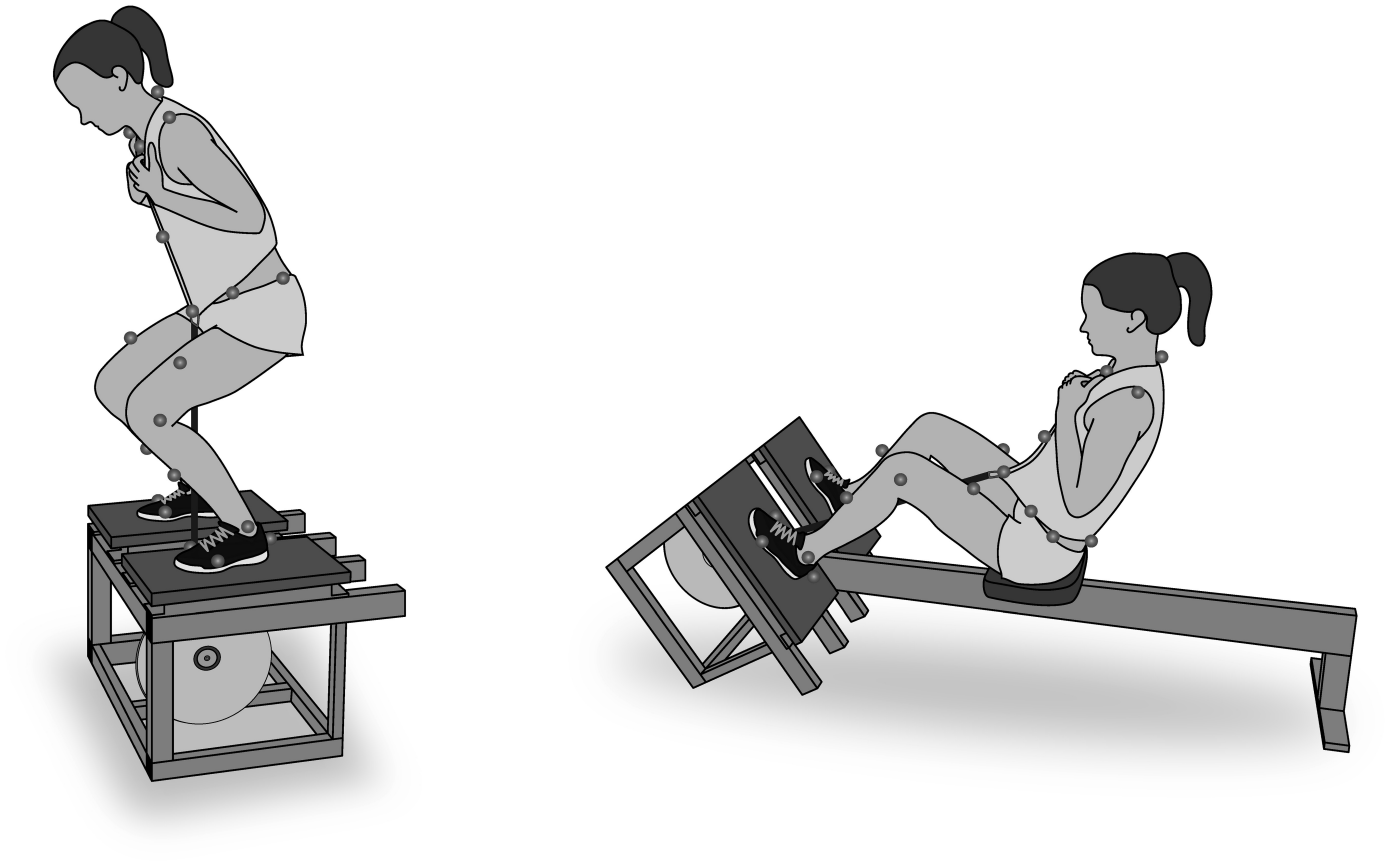
Schematic illustration of the squat performed standing on the flywheel exercise device, and the leg press, performed seated on a sliding seat using the flywheel exercise device. A strap connects the participant to the flywheel shaft through a harness.

During the experimental session, each subject warmed up and then performed one set of five maximal repetitions of the squat and leg-press exercises, respectively, using a custom-made flywheel-exercise device. Each set of five maximal repetitions was preceded by two submaximal repetitions to gain momentum in the flywheel. During both exercises, each subject kept the arms crossed over the chest and was instructed to aim for a maximum of 90° flexion of the knees. Each subject rested at least 5 min between sets to allow for full recovery and to prevent fatigue. The order of the squat and leg-press exercise trials was randomised among subjects. Five flywheels with a total moment of rotational inertia of 0.125 kg·m^2^ were used and the flywheel load was transmitted from the flywheel device to the subject via a strap attached to a harness (Figure 1).

The squat was performed with a vertical movement standing upright on the flywheel box whereas the leg press was performed horizontally while on a sliding seat with the feet positioned on the inclined flywheel box (Figure 1). The stance width was chosen by the subjects individually within the margins of the flywheel box, but they were asked to adjust the anteroposterior foot position so that the flywheel strap was positioned near the midfoot.

### Motion Analysis and Musculosketal Modelling

Both exercise trials were performed while collecting three-dimensional marker trajectory data by an eight-camera marker-based motion-capture system (ProReflex MCU240, Qualisys AB, Gothenburg, Sweden) at a sampling frequency of 100 Hz (cf. Lorenzetti et al., 2017). Retroreflective markers were placed bilaterally over the following bony landmarks: acromion, anterior- and posterior superior iliac spine, iliac crest, lateral femoral epicondyle, lateral malleolus, calcaneus, and first- and fifth metatarsal head. In addition, markers were placed on the spinous process of the seventh cervical vertebra, in the cavity between the two clavicular bones, and on the frontal part of the thighs and shins. Two force plates (Bertec Corp., Columbus, OH, USA; sampling rate 2000 Hz) were mounted onto the flywheel exercise device (Figure 1) to measure the ground reaction forces and moments (GRF&M) from each foot. Although not actually measuring the reaction force on the ground, but rather the contact force between the feet and the exercise device, the force plate measurements are henceforth referred to as GRF to use a well-established term. Flywheel force was registered by a linear transducer (Burster, Gernsbach, Germany; sampling rate 2000 Hz) connected between the harness and flywheel strap. Motion and force data were simultaneously collected and later processed (Qualisys Track Manager, version 2.13, Qualisys).

The experimentally collected data were then imported to a musculoskeletal model (AnyBody Modelling System, v.7.0.1, AnyBody Technology A/S, Aalborg, Denmark), where joint kinematics and kinetics as well as muscle forces were computed through inverse dynamics and optimisation. Marker trajectory and force data were filtered using a fourth-order, zero-phase low-pass Butterworth filter with cut-off frequencies of 6 Hz and 12 Hz, respectively. The musculoskeletal model used was a full-body model (AnyMoCap, AMMR v.1.6.6, Lund et al., 2017), consisting of a leg model (Klein Horsman et al., 2007) and a lumbar spine model (de Zee et al., 2007). The model had 21 degrees of freedom and contained 347 muscle-tendon units (MTUs). Three-element Hill-type muscle models were included for the legs, while the lumbar spine had simple muscle models. Segment lengths and marker positions were identified using the data from a standing static trial (Andersen et al., 2010). Further individual fitting of the model was done through linear scaling under a sex-specific length-mass-fat scaling criterion (Frankenfield et al., 2001; Rasmussen et al., 2005). Joint kinematics of the dynamic trials were then computed by minimising the least-square differences between experimental and model marker positions (Andersen et al., 2009). The sum of muscle activities cubed were minimised through a muscle recruitment algorithm, i.e., a third order polynomial objective function (Damsgaard et al., 2006), to resolve the indeterminacy of the problem. The pelvis-seat contact during the leg-press trials was simulated by moving the vertical component of the ground residuals from the torso to the pelvis. A more detailed description of the musculoskeletal model can be found in (Sjöberg et al., 2020).

### Data Processing

Total exercise load was assessed by studying the magnitude of the resultant GRF (rGRF) from both feet. Lower-extremity sagittal plane joint angles and NJMs from the right leg were analysed. Joint angles were expressed as the relative angle between the local coordinate systems of articulating segments. Events that demarcated each exercise repetition were defined by the knee flexion angle, starting at minimum knee flexion (i.e., knee extension), through the eccentric phase ending at maximum knee flexion, then the concentric phase ending again at minimum knee flexion.

The muscles analysed were plantar flexors: soleus and gastrocnemius; knee extensors: vastus lateralis, vastus medialis, vastus intermedius, and rectus femoris; and hip extensors: gluteus maximus, adductor magnus, and biceps femoris. Each muscle was represented by several musculotendon units (MTUs) in the musculoskeletal model and the output variables studied were force and activity, the latter being force in relation to the estimated maximal force-generating capacity (i.e., strength) of the MTU. The force from each muscle was computed as the sum of forces from all MTUs whereas activity, which is a relative measure, was averaged for all MTUs of the corresponding muscle. To allow for inter-subject comparisons, forces were normalised to each subject's body weight and NJMs were normalised to body weight and body height.

All variables were averaged over three consecutive repetitions from each trial, including the repetition with the highest rGRF. Peak joint angles were analysed. External forces, NJMs, muscle forces and muscle activities were analysed during the most demanding phase by computing the average of the time period starting from 15° before maximum knee flexion and ending at 15° after maximum knee flexion. This is considered the most demanding phase of the repetition since the flywheel is decelerated to a full stop during the late phase of the eccentric action (within the final 15° of flexion), and then mainly accelerated during the subsequent initiation of the concentric action (within the initial 15° of extension). Comparisons were also made between eccentric and concentric phases during this period, wherein mean values were calculated for data spanning the last 15° before maximum knee flexion and the first 15° after maximum knee flexion, respectively. This data processing was performed using commercially available software (Matlab R2018b, The MathWorks Inc., Natick, MA, USA).

### Statistical Analyses

Analyses of intra-subject differences between exercise modalities were performed using paired samples *t*-tests. Eccentric-concentric differences were analysed using a two-way repeated measures ANOVA (Exercise Type × Exercise Phase/Joint). Sphericity was assessed by Mauchly's test and the Greenhouse-Geiser correction was used to adjust the degrees of freedom, if necessary. Significant main effects revealed by the analysis of variance were further studied with pairwise comparisons using Tukey's HSD *post-hoc* test and the significance level was set at α = 0.05. Furthermore, effect sizes (Cohen's *d*) were calculated using a 95% confidence interval and pooled estimates of SD. Effect sizes of 0.2–0.5 were interpreted as small, 0.5–0.8 as moderate, and >0.8 as large. All statistical tests were computed using commercially-available software (Statistica 8.0, StatSoft, Tulsa, OK, USA and Excel 2016, Microsoft Corp., Redmond, WA, USA) and results are presented as means ± SD.

## Results

Subjects generated a slightly larger rGRF in squat (2.20 ± 0.34 BW) than in leg press (2.04 ± 0.43 BW, *p* = 0.04, d = 0.42). By contrast, the flywheel force was greater in leg press (2.01 ± 0.43 BW) than in squat (1.18 ± 0.33 BW, *p* < 0.001, d = 2.19). The mean time period of the most demanding phase was shorter for leg press than for squat (1.72 ± 0.20 s and 2.25 ± 0.36 s, respectively, *p* < 0.001, d = 1.82).

Subjects reached a greater peak knee-flexion angle in leg press (*p* = 0.01), while the peak ankle-dorsiflexion angle was greater in squat (*p* = 0.004). Similar peak hip-flexion angles were obtained in both exercises (*p* = 0.51; Table 2).

**Table 2:** Peak-joint angles for leg press and squat.

**Variable**	**Leg Press**	**Squat**	***d***
Ankle dorsi-flexion (degrees)	32 ± 12^**^	42 ± 8	1.01
Knee flexion (degrees)	109 ± 9^**^	98 ± 5	1.56
Hip flexion (degrees)	78 ± 12	81 ± 13	0.28

*Values are mean ± SD. Significantly different from squat*:

** *p ≤ 0.01*.

*d—Cohen's effect size (small: 0.2–0.5, moderate: 0.5–0.8, strong: >0.8)*.

**Figure 2:**
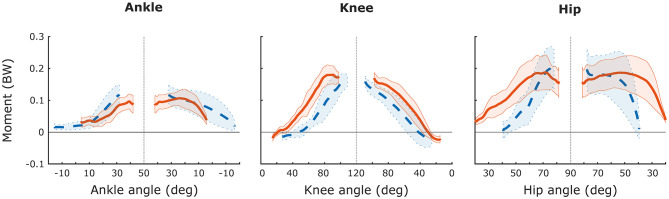
Net-joint moments for the ankle, knee, and hip in relation to joint angle during leg press (dashed blue line) and squat (solid orange line). Moments are normalised to body weight in newtons (BW). Curves represent group mean values and shaded areas are ±1 SD. Vertical dashed line indicate transition from the eccentric to the concentric phase.

**Figure 3:**
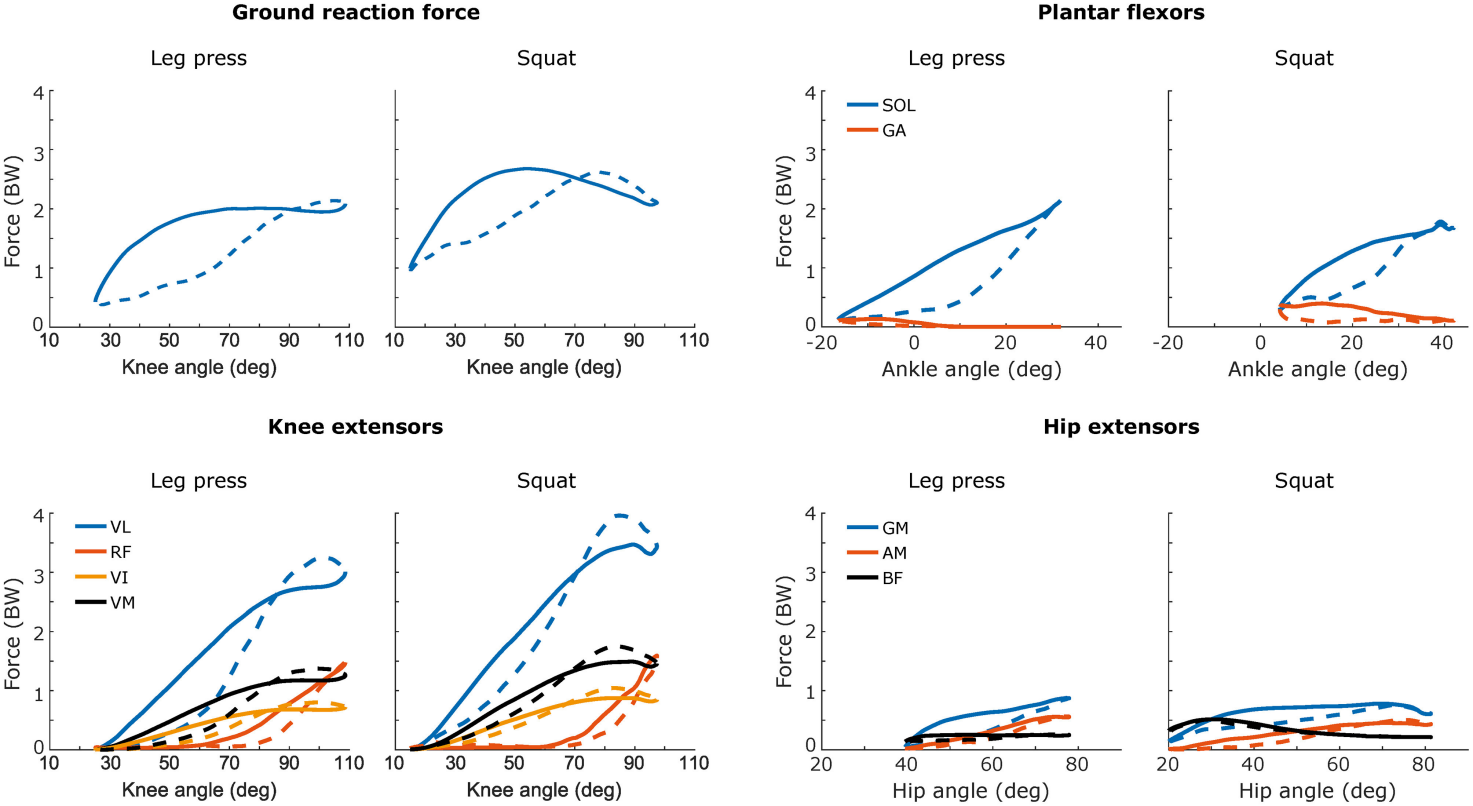
Resultant ground reaction force and knee extensor muscle forces as a function of knee flexion angle (left), and ankle plantar-flexor muscle forces of ankle-dorsiflexion angle and hip extensor muscle forces of hip flexion angle (right) during the eccentric (dashed) and the concentric (solid) phases of leg press and squat. *VL: vastus lateralis, RF: rectus femoris, VI: vastus intermedius, VM: vastus medialis, SOL: soleus, GA: gastrocnemius, GM: gluteus maximus, AM: adductor magnus, BF: biceps femoris*. Forces are normalised to body weight (BW) and curves show group mean values. n = 9 for all variables except for GM force where *n* = 7.

Knee-extension NJM (*p* = 0.005) and vastii muscle forces were greater in squat than in leg press (*p* < 0.007; Table 3, Figures 2, 3). Hip-extension NJM was moderately greater in leg press (*p* = 0.03); simultaneously, the gluteus maximus muscle force showed a tendency of being larger during leg press than squat (*p* = 0.06, Table 3). Ankle-plantarflexion NJM was similar in the two exercises (*p* = 0.16, Table 3).

**Table 3:** Net-joint moments and muscle forces for leg press and squat during the most demanding phase, spanning from 15° before to 15° after maximum knee-flexion angle.

**Variable**	**Leg Press**	**Squat**	***d***
Ankle plantar-flexion moment (BW)	0.11 ± 0.028	0.091 ± 0.023	0.72
Knee-extension moment (BW)	0.15 ± 0.019^**^	0.17 ± 0.018	0.97
Hip-extension moment (BW)	0.19 ± 0.059^*^	0.16 ± 0.042	0.56
VL force (BW)	3.0 ± 0.66^**^	3.5 ± 0.60	0.92
VI force (BW)	0.72 ± 0.16^**^	0.89 ± 0.15	1.06
VM force (BW)	1.3 ± 0.27^**^	1.5 ± 0.26	1.00
RF force (BW)	1.2 ± 0.94	1.2 ± 0.45	0.05
BF force (BW)	0.25 ± 0.10	0.22 ± 0.072	0.39
GM force^†^ (BW)	0.88 ± 0.17	0.73 ± 0.14	1.01
AM force (BW)	0.55 ± 0.19	0.45 ± 0.20	0.50
SOL force (BW)	2.0 ± 0.40	1.7 ± 0.72	0.47
GAS force (BW)	17 × 10^−12^ ± 2.1 × 10^−12^	0.12 ± 0.27	0.60

*Moments and forces are normalised to body weight in newtons (BW). Values are mean ± SD. VL, vastus lateralis muscle; VI, vastus intermedius muscle; VM, vastus medialis muscle; RF, rectus femoris muscle; BF, biceps femoris muscle; GM, gluteus maximus muscle; AM, adductor magnus muscle; SOL, soleus muscle; GAS, gastrocnemius muscle*.

*Significantly different from squat*:

* *p ≤ 0.05*,

** *p ≤ 0.01*.

*d—Cohen's effect size (small: 0.2–0.5, moderate: 0.5–0.8, strong: >0.8)*.

† *n = 7*.

**Figure 4:**
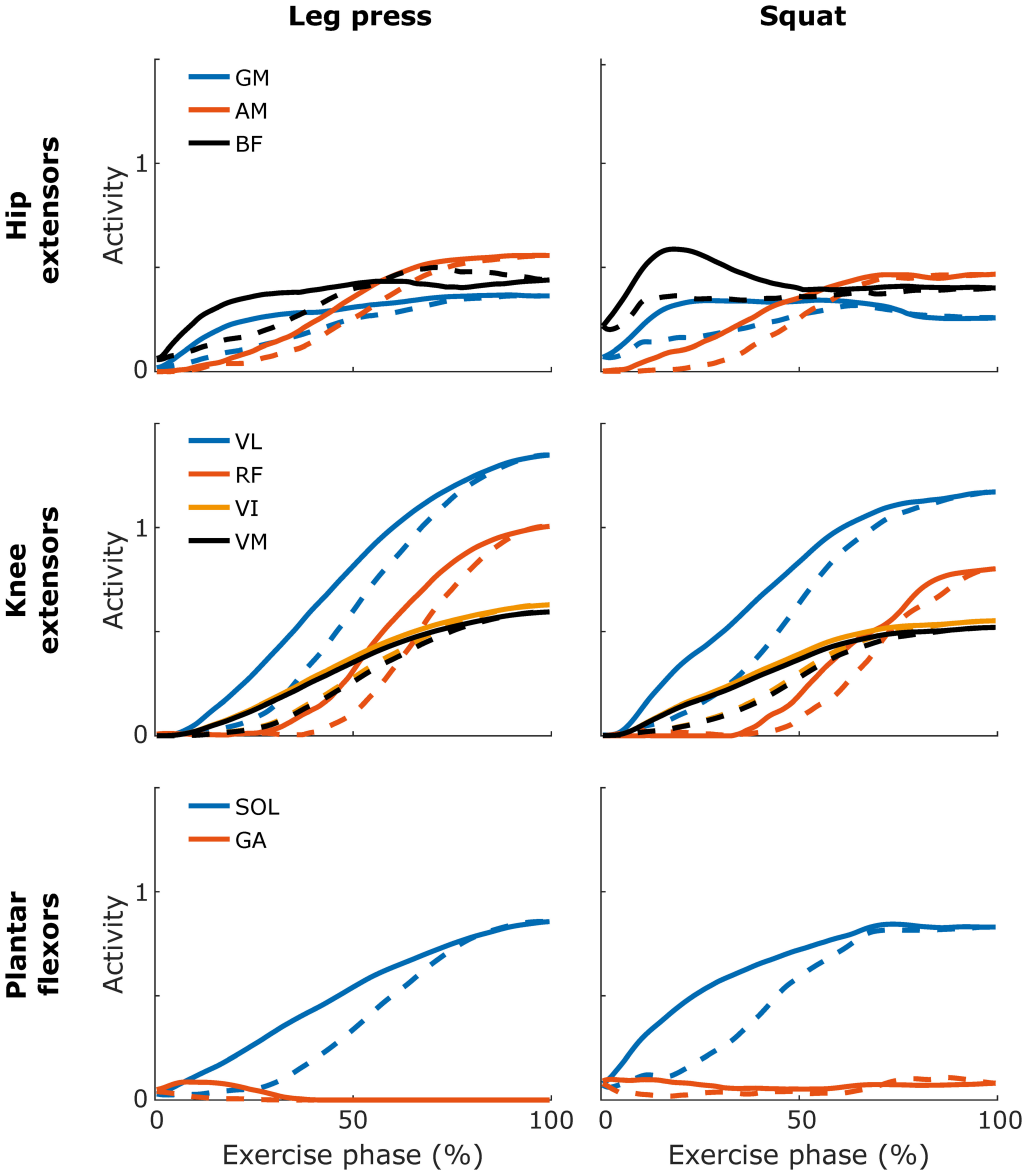
Hip extensor, knee extensor, and ankle plantar-flexor muscle activity as a function of time-normalised exercise phase for eccentric (dashed) and concentric (solid) parts of leg press and squat. *GM: gluteus maximus, AM: adductor magnus, BF: biceps femoris, VL: vastus lateralis, RF: rectus femoris, VI: vastus intermedius, VM: vastus medialis, SOL: soleus, GA: gastrocnemius*. Curves show group mean values. n = 9 for all variables except for gluteus maximus activity where n = 7.

Muscle activities did not differ significantly between exercises, although the hip extensor activities tended to be greater in leg press than squat (gluteus maximus: *p* = 0.10, d = 1.21; adductor magnus: *p* = 0.08, d = 0.71, Figure 4). Muscles almost invariably showed maximum activity shortly before and after the eccentric-concentric transition.

Evaluating the eccentric versus the concentric actions, all vastii muscle forces were slightly to moderately greater during the eccentric than the concentric action (vastus lateralis force: F = 11.2, *p* = 0.01, η^2^ = 0.58, vastus medialis force: F = 12.6, *p* = 0.007, η^2^ = 0.61, vastus intermedius force: F = 14.1, *p* = 0.006, η^2^ = 0.64), i.e., eccentric overload, for both exercises (Figure 3; Table 4). The rectus femoris showed slightly to moderately greater activity during the concentric than eccentric phase (F = 19.1, *p* = 0.002, η^2^ = 0.70), in both squat (*p* = 0.006) and leg press (*p* = 0.003, Table 4). Similarly, the gluteus maximus activity was overall slightly greater during the concentric than the eccentric phase (F = 9.5, *p* = 0.02, η^2^ = 0.61), but not different during squat (*p* = 0.26) or leg press (*p* = 0.07, Table 4).

**Table 4:** Muscle force and muscle activity during eccentric (Ecc) and concentric (Con) phases of leg press and squat.

	**Leg press**			**Squat**		
**Variable**	**Ecc**	**Con**	***d***	**Ecc**	**Con**	***d***
VL force (BW)	3.1 ± 0.73^*^	2.8 ± 0.65	0.42	3.7 ± 0.62^*^	3.4 ± 0.62	0.50
VI force (BW)	0.76 ± 0.17^*^	0.69 ± 0.16	0.45	0.94 ± 0.16^**^	0.85 ± 0.16	0.55
VM force (BW)	1.3 ± 0.30^*^	1.2 ± 0.27	0.43	1.6 ± 0.28^**^	1.5 ± 0.27	0.53
RF force (BW)	1.2 ± 1.1	1.3 ± 0.84	0.07	1.1 ± 0.39	1.3 ± 0.52	0.37
BF force (BW)	0.25 ± 0.10	0.25 ± 0.10	0.09	0.22 ± 0.070	0.22 ± 0.076	0.00
GM force^†^ (BW)	0.85 ± 0.15	0.91 ± 0.20	0.37	0.71 ± 0.13	0.75 ± 0.15	0.27
AM force (BW)	0.55 ± 0.17	0.55 ± 0.21	0.03	0.47 ± 0.20	0.44 ± 0.20	0.16
SOL force (BW)	2.0 ± 0.46	2.0 ± 0.40	0.03	1.7 ± 0.75	1.7 ± 0.72	0.03
GAS force (BW)	0.00 ± 0.00	0.00 ± 0.00	0.00	0.11 ± 0.25	0.12 ± 0.30	0.04
VL activity	1.2 ± 0.27	1.2 ± 0.21	0.36	1.1 ± 0.19	1.1 ± 0.19	0.28
VI activity	0.54 ± 0.13	0.58 ± 0.11	0.31	0.50 ± 0.094	0.53 ± 0.095	0.24
VM activity	0.51 ± 0.13	0.55 ± 0.10	0.32	0.47 ± 0.090	0.50 ± 0.091	0.24
RF activity	0.75 ± 0.49^**^	0.89 ± 0.43	0.30	0.56 ± 0.15^**^	0.66 ± 0.15	0.65
BF activity	0.47 ± 0.38	0.43 ± 0.34	0.13	0.39 ± 0.21	0.40 ± 0.22	0.07
GM activity^†^	0.34 ± 0.033	0.38 ± 0.054	0.74	0.29 ± 0.060	0.31 ± 0.058	0.37
AM activity	0.50 ± 0.10	0.54 ± 0.13	0.34	0.44 ± 0.086	0.46 ± 0.090	0.19
SOL activity	0.73 ± 0.26	0.79 ± 0.22	0.21	0.80 ± 0.27	0.83 ± 0.28	0.09
GAS activity	0.00 ± 0.00	0.00 ± 0.00	0.23	0.078 ± 0.20	0.074 ± 0.20	0.02

*Forces are normalised to body weight in newtons (BW). Values are mean ± SD for the most demanding phase, spanning 15° before and 15° after maximum knee-flexion angle for Ecc and Con, respectively. VL, vastus lateralis muscle; VI, vastus intermedius muscle; VM, vastus medialis muscle; RF, rectus femoris muscle; BF, biceps femoris muscle; GM, gluteus maximus muscle; AM, adductor magnus muscle; SOL, soleus muscle; GAS, gastrocnemius muscle*.

*Significantly different from Con*:

* *p ≤ 0.05*,

** *p ≤ 0.01*.

*d—Cohen's effect size (small: 0.2–0.5, moderate: 0.5–0.8, strong: > 0.8)*.

† *n = 7*.

## Discussion

The aim was to compare the biomechanical loading patterns in flywheel leg press and squat. The results should be viewed in the context that, while targeting the same muscle groups, the executions of these two exercises have some fundamental differences. During squat, the subject had to maintain balance, as opposed to the leg press, during which the subject's trajectory was guided and the body weight was supported by the sliding seat. Furthermore, in leg press, virtually all force produced by the muscles was used to accelerate and decelerate the flywheel, whereas in squat the muscle force was, besides rotating the flywheel, required to lift and lower the body weight, explaining the almost two-fold higher flywheel force and shorter repetition duration in leg press than in squat.

During the most demanding phase, when the flywheel was decelerated to a full stop and then accelerated again, a greater load about the hip joint was observed in leg press than squat, as indicated by a moderately higher hip-extension NJM in leg press. There was a tendency of greater gluteus maximus activity and force production in leg press, but it did not reach statistical significance. The greater hip load during leg press emphasises the significance of the substantially larger flywheel force applied to the upper body. Bryanton et al. (2012), similarly found that hip NJM increases with increased external barbell load during back squat. Conversely, the load about the knee joint was higher during flywheel squat than leg press, as indicated by greater knee-extension NJM and force production in the vastii muscles. The overall relative muscle activity of the knee extensors was, however, similar in squat and leg press. No significant difference was seen in the ankle-plantarflexion NJM between the two exercises, although a moderate effect size was present. A comparison of the flywheel squat and leg press at equal submaximal load has previously shown greater plantarflexion NJM in leg press than squat (Sjöberg et al., 2020), which aligns with the trend of the present data.

### Overall Muscle Use and Joint Loadings During Squat and Leg-Press Exercise

As mentioned, all vastii muscles showed considerably higher force production during squat than leg press (Table 3), and the total exercise load (rGRF) was slightly greater in squat than leg press. Most likely, this is attributable to the smaller knee-flexion angle attained during squat than during leg press (Table 2), as maximum voluntary knee-extension force increases profoundly and linearly with decreasing knee-flexion angle (Eloranta and Komi, 1980; Kulig et al., 1984). The question arises as to why subjects virtually invariably attained greater knee-flexion angles in leg press than in squat, despite a targeted 90° in both exercises. It can be argued that the high exercise load was not a key factor, since the same pattern was observed during submaximal flywheel exercise (Sjöberg et al., 2020). One can speculate that the removed need to maintain balance while seated on a sliding seat, as well as the greater plantarflexion angle in the leg press due to the angled force plate positions, particularly during the high kinetic energy during the late eccentric phase, may have contributed to the subjects' spontaneously higher knee-flexion angle in leg press (Sjöberg et al., 2020). From a safety perspective, the classic notion is that quadriceps resistance exercise should not be performed at knee flexions deeper than 90° so as to avoid presumed high compressive and shear forces that may damage knee ligaments (Escamilla, 2001). More recent reviews (Hartmann et al., 2013) and experimental studies (Bryanton et al., 2012) do not, however, support this notion. By contrast, the implicit lower external load at deep squat or leg press, in combination with load distribution over a larger area beneath the quadriceps tendon and patella (“wrapping effect”), rather support such routines in individuals without joint disorders (Hartmann et al., 2013).

The findings of higher total load during squat and a propensity for deeper knee flexion and higher contraction velocity during leg press should also be viewed in a training efficacy perspective. Resistance training studies comparing squats performed at different knee-joint angles do not advocate the exercise type that allows the heaviest load to be lifted; instead, a deep rather than shallow knee-inflexion point appears to be a key factor for strength development and hypertrophy of the quadriceps muscle during squat training regimens (Bryanton et al., 2012; Bloomquist et al., 2013; Hartmann et al., 2013; Kubo et al., 2019). Greater muscle-tendon forces over the knee joint and longer knee-extensor muscles have been postulated as the main stimuli for these increments (Bryanton et al., 2012; Bloomquist et al., 2013). Thus, for strength training of the quadriceps muscles, flywheel leg press might be preferable to flywheel squat, despite present evidence of higher total load in squat. This notion was supported by our finding that the calculated vastii muscle activities did not differ between leg press and squat despite greater force output and, hence, greater total load in squat. Thus, taking the force-length and force-velocity relationships into account in biomechanical analyses of strength training effects concerts with the reasoning that the relative muscular effort is important for resistance exercise efficacy (Chiu, 2018).

The averaging or summing of kinematic measures was introduced to calculate the total mechanical output of the lower limb during gait, jumping or lifting (Flanagan and Salem, 2008). During back squats, average NJM and work have been reported to be consistently larger about the hip than the knee and ankle joints (Flanagan and Salem, 2008; Bryanton et al., 2012). When the external barbell load approached the individual's maximum, a common strategy was to further increase hip and ankle NJMs while moving the centre of pressure (COP) anteriorly towards the forefoot (Flanagan and Salem, 2008). Likewise, Chiu and Salem (2006) showed that average hip extensor NJM was greater than average knee and ankle NJM during flywheel front squats. However, in both aforementioned studies (Chiu and Salem, 2006; Flanagan and Salem, 2008), the NJM was averaged over the entire movement, whereas in the present study, the NJM was averaged around the eccentric-concentric turning point (±15°), thus rather describing near maximum NJM. These estimates implied similar knee-joint as hip-joint NJM during the flywheel squat exercise, whereas a greater hip- than knee-joint NJM would have been attained, had the NJMs been averaged over the entire ranges of motion (Figure 2). Gullett et al. (2009) reported that the maximum knee-extension NJM was greater during back than front squats, but did not report on hip or ankle NJM. The results of Gullett et al. (2009), and the current use of a harness, distributing the load to the shoulders and the back, suggests that the present flywheel squat resembles a conventional barbell back squat rather than a front squat.

### Concentric, Eccentric, and Specific Muscle Actions

The resistance of the flywheel inertia allows generation of maximal force throughout the concentric muscle actions. By contrast, the decelerating phase with eccentric muscle actions typically occurs over a shorter duration, near the turning point where eccentric overload may be produced. Accordingly, the vastii muscle force estimates indicated small and moderate eccentric overload for leg press and squat, respectively (Figure 3). Our results did, however, not indicate eccentric overload in the hip extensors, plantar flexors or in the rectus femoris muscle during squat or leg press. Previous flywheel studies demonstrated substantial eccentric overload during open-loop knee extensions (Norrbrand et al., 2010), whereas in closed-loop leg-extension exercises, eccentric overload was not always apparent (Alkner and Tesch, 2004; Norrbrand et al., 2011). One explanation for the generally modest eccentric overload in the present and previous flywheel leg-extension studies may be that concentric peak rGRF values occur at relatively extended knee and hip angles where muscle mechanics are favourable, i.e., the muscles are near the lengths at which they produce the most active force, whereas the peak eccentric rGRF is produced in the unfavourable flexed position approaching the turning point (Figure 3). In addition, the shares of concentric and eccentric activity contributed by these muscle groups differ during the course of a given cycle. Since the leg press and squat exercises activate multiple muscle groups about several joints, the timing of muscle activity may differ. Thus, in the present experiments while knee extensors showed clear eccentric overload, plantar flexors were mostly active during concentric actions. Similar patterns have been described for jumping and skating activities (Houdijk et al., 2000). Conceivably, the dynamic loading of flywheel inertia allowing some flexibility of muscle activation and joint loading, may closer mimic certain locomotory and sports activities than the isotonic loading of conventional weightlifting (Berg et al., 1995; Houdijk et al., 2000).

Inevitably, as muscles are stronger eccentrically than concentrically (Eloranta and Komi, 1980), traditional weight training providing constant load results in submaximal eccentric actions, as typically indicated by EMG recordings (Adams et al., 1992). Thus, the greater capacity of a muscle to produce force during eccentric than concentric actions implies that, for the same load, the relative muscle activity would be lower eccentrically than concentrically. On the contrary, our results reveal similar relative activities in the eccentric and concentric phases in all muscles, except for the rectus femoris, which showed slightly and moderately greater concentric activity in leg press and squat, respectively (Table 4, Figure 4). These results support the notion that both the squat and leg press exercise induced prominent eccentric muscle actions, despite that the force estimates indicated no or modest eccentric overloading. It has been suggested that it is the demanding eccentric muscle actions that contribute to the muscle-injury prevention (Askling et al., 2003; de Hoyo et al., 2015), and the extensive training response, i.e., strength increase, sprint improvement, and hypertrophy (cf. Maroto-Izquierdo et al., 2017), following concentric-eccentric flywheel resistance exercises. Although there are indications that responses to flywheel resistance training may be beneficial compared to those of weight-stack or free-weight training (Maroto-Izquierdo et al., 2017; Petré et al., 2018), specific training studies comparing long-term responses of, flywheel and e.g., barbell-squat resistance training, are warranted.

The quadriceps muscle force patterns in squat and leg press were similar to those observed in deadlift and split squat (Schellenberg et al., 2017), although when comparing the relative force outputs in the different quadriceps muscles, both flywheel exercises seemed to activate the rectus femoris to a greater extent than do the dead lift and the spilt squat (cf. Schellenberg et al., 2017). This notion is in line with an earlier study comparing use of rectus femoris during flywheel and barbell squat (Norrbrand et al., 2011). While the vastii muscles were active during most of the range of motion, the rectus femoris was only activated during greater knee-flexion angles where the knee-extensor load was maximal. The dual function of the rectus femoris, acting both as knee extensor and hip flexor, is likely the reason for the different recruitment patterns. Similarly, the biceps femoris, the main antagonist of the rectus femoris, is a biarticular muscle and acts as both knee flexor and hip extensor. During the late eccentric phase, the rectus muscle was highly active and the biceps femoris was moderately active (Figure 4); the concomitant activation of these antagonistic muscles presumably served to stabilise the knee and hip joints during the high-loaded turning phase from eccentric to concentric action. During the late concentric portion of the squat, by contrast, the biceps femoris muscle force and activity increased promptly, whereas the rectus femoris showed no activity (Figure 4), suggesting that, during this sequence of the motion, with relatively extended knees, the biceps acted exclusively as a hip extensor, with minimal antagonistic interference from the rectus muscle. Such recruitment pattern of the biceps femoris was not observed during the leg press. A similar late prominent, concentric activation of the hamstrings and other hip extensors, has previously been noted during maximal barbell back squat, as evidenced by EMG recordings (Yavuz and Erdag, 2017); knee-extensor EMG, by contrast, peaked at the turning point or during the late eccentric phase. The activation of hip extensors during the late concentric phase was linked to an increased forward lean of the upper body at maximum load, and the authors advocated proper lifting technique to avoid a lumbar injury (Yavuz and Erdag, 2017).

The soleus muscle was the main contributor to plantarflexion (Figure 4), which agrees with results from Toutoungi et al. (2000). In the most demanding phase of the repetition when the knee is in deep flexion, the gastrocnemius is relatively weak, as it is very short. It is reasonable to state that greater recruitment of soleus than gastrocnemius is a more “economical” means to provide plantarflexion moment. Furthermore, the biarticular gastrocnemius is also a knee flexor and its activity in this phase would place more demand on the already maximally-activated knee extensors. The musculoskeletal model in general finds the most “economical” solution in terms of muscle activation, and thus does not favour co-contraction; therefore, a certain underestimation of muscle activity might occur where antagonistic muscle activity is prominent and may thus partly explain why the low computed gastrocnemius activity does not entirely agree with experimental EMG results from free-weight squat and leg press (Escamilla et al., 1998). Moreover, higher experimentally-observed co-contraction has been reported in free-weight squat than in leg press (Escamilla et al., 2001). Dynamic balance maintenance is more challenging in squat, but musculoskeletal modelling generally cannot capture such effects fully. Despite these limitations in predicting absolute values, modelling outputs are generally valid for inter-condition comparisons.

### Methodological Considerations

Despite a targeted 90-degree knee-flexion angle in both exercises, the subjects performed leg press with a greater angle, which, as mentioned, partly may have been caused by the anterior tilt of the footplate position in this condition. On the one hand, the different knee-flexion angles in the two exercise modes have somewhat complicated the present comparison between squat and leg press. On the other hand, the subject's tendency of spontaneously choosing deeper knee flexion in leg press than squat may be an important observation, since it can be presumed that a deeper knee flexion is beneficial from a strength-training perspective (Bloomquist et al., 2013; Hartmann et al., 2013).

The motion capture data were sampled at 100 Hz, which may be in the low frequency spectrum for capturing several exercise movements. However, the sampling frequency was presumably adequate, considering that the movement velocities of the present flywheel resistance exercises were very slow (repetition duration; leg press = 2.51 ± 0.31 s, squat = 3.20 ± 0.50 s), and that in previous studies concerning motion capture data during squat exercises, 100 Hz sampling rate has been standard (cf. Lorenzetti et al., 2017). In addition, a very high sampling frequency may cause problems with marker identification.

## Conclusion

Present results revealed that the quadriceps muscle activity was similar in maximal flywheel squat and leg press, while rGRF and knee-extension NJM were greater in squat than leg press. Together with the finding of a propensity to perform leg press at deeper knee-flexion angle than squat, this suggests that leg press is the preferable leg-extensor resistance exercise, both from a training efficacy and an injury risk perspective. It remains to be investigated, however, whether the presumed greater lumbar loading in leg press, as reflected by greater hip-extension NJM, is a desirable added training stimulus or constitutes a risk of inflicting back injury.

## Data Availability Statement

The raw data supporting the conclusions of this article will be made available by the authors, without undue reservation.

## Ethical Statement

The experimental protocol conformed to the Declaration of Helsinki and was approved by the Stockholm Regional Human Ethics Committee (Dnr 2016/459-31/2).

## Author Contributions

MS, OE, HB, EG-F, and PS designed the study. MS and LN performed the data collection. MS and MA tailored the musculoskeletal model. MS performed data processing and analyses and drafted the manuscript together with OE, HB, and LN. All authors critically reviewed and agreed to the manuscript.

## Conflict of Interest

The authors declare that the research was conducted in the absence of any commercial or financial relationships that could be construed as a potential conflict of interest.

## Publisher's Note

All claims expressed in this article are solely those of the authors and do not necessarily represent those of their affiliated organizations, or those of the publisher, the editors and the reviewers. Any product that may be evaluated in this article, or claim that may be made by its manufacturer, is not guaranteed or endorsed by the publisher.
